# Web Queries as a Source for Syndromic Surveillance

**DOI:** 10.1371/journal.pone.0004378

**Published:** 2009-02-06

**Authors:** Anette Hulth, Gustaf Rydevik, Annika Linde

**Affiliations:** Department of Epidemiology, Swedish Institute for Infectious Disease Control, Solna, Sweden; U.S. Naval Medical Research Center Detachment/Centers for Disease Control, United States of America

## Abstract

In the field of syndromic surveillance, various sources are exploited for outbreak detection, monitoring and prediction. This paper describes a study on queries submitted to a medical web site, with influenza as a case study. The hypothesis of the work was that queries on influenza and influenza-like illness would provide a basis for the estimation of the timing of the peak and the intensity of the yearly influenza outbreaks that would be as good as the existing laboratory and sentinel surveillance. We calculated the occurrence of various queries related to influenza from search logs submitted to a Swedish medical web site for two influenza seasons. These figures were subsequently used to generate two models, one to estimate the number of laboratory verified influenza cases and one to estimate the proportion of patients with influenza-like illness reported by selected General Practitioners in Sweden. We applied an approach designed for highly correlated data, *partial least squares regression*. In our work, we found that certain web queries on influenza follow the same pattern as that obtained by the two other surveillance systems for influenza epidemics, and that they have equal power for the estimation of the influenza burden in society. Web queries give a unique access to ill individuals who are not (yet) seeking care. This paper shows the potential of web queries as an accurate, cheap and labour extensive source for syndromic surveillance.

## Introduction

In the field of *syndromic surveillance*, various sources are exploited for outbreak detection, monitoring and prediction. The assumption is that by systematically collecting and analysing data such as school absenteeism [1], over-the-counter pharmaceutical sales [2], [3], ambulance dispatch data [4], [5], or emergency department chief complaints [6], [7] and medical diagnoses [8], illness clusters may be detected earlier than by conventional surveillance. An earlier detection will, in turn, allow for interventions that are assumed to lower the morbidity and mortality resulting from the outbreak. Syndromic surveillance can also play an important role in monitoring the impact and the geographical spread of an infection, both for unexpected outbreaks, as well as for regularly occurring infections, such as influenza.

To our knowledge, no evaluation regarding syndromic surveillance has been performed on queries submitted to medical web sites, although this is assumed to be a useful source [9], [10]. Since the first submission of this paper, two studies on general purpose search engine queries have been published; one based on Google search queries [11] and one on Yahoo! search queries [12]. Studies in England [13] and Canada [14] using data from medical telephone help lines were performed, suggesting that such data may have a great potential to early warning systems. Johnson et al. examined access of influenza-related articles on a medical web site in the United States [10]. A Finnish study described the correlation between searches in a reference database by medical doctors and laboratory reports sent to the National Infectious Disease Register in Finland [15]. In the experiments, logs showing which pages were visited for three investigated diseases were collected from the users' computers. The conclusion drawn was that database searches may provide a valuable tool in early detection.

According to Statistics Sweden (www.scb.se), 64 per cent of the male and 53 per cent of the female Swedish population use the internet on a daily basis (first quarter 2007). During the same period, 21 per cent of the male and 30 per cent of the female population searched for health related information. An example of a medical web site is Vårdguiden (www.vardguiden.se), which is owned by the Stockholm County Council and established in 2002. The web site provides information on various aspects of health care, among those self-help advice and preventive health advice. In March 2008, the web site had 800 000 visitors and during 2007, three million queries were submitted to the search engine on the web site.

This paper describes an investigation of the feasibility of web queries as a source for syndromic surveillance. For this first case study, we selected influenza. Each year, a large number of people are thought to be infected by influenza. However, little is known of the true burden of illness nationally and globally. Two approximations are currently used at the Swedish Institute for Infectious Disease Control: laboratory verified influenza diagnoses from all laboratories diagnosing influenza in Sweden and the percentage of cases with influenza-like illness (ILI) reported through a sentinel system [16]. These two data sets have also been used for prediction of timing and height of the influenza peak [17]. However, both sources demand that the cases seek medical care, and there may be an over-representation of groups that are vulnerable for severe disease in the reporting. There is also a risk that the sentinel system collapses during a pandemic, since the health care staff will be overloaded with patient care. In addition, it is a rather expensive and time consuming system to maintain. It is therefore important to find other sources of information.

In the described retrospective study, we analysed search logs collected for the above mentioned Vårdguiden. The number of queries related to influenza was calculated for two influenza seasons (2005/2006 and 2006/2007). These numbers were subsequently used to generate two estimation models, one for the number of laboratory verified cases and one for ILI reported in Sweden for the same two periods. The hypothesis of the described study was that queries on influenza submitted to the web site would provide a basis for the estimation of the timing of the peak and the intensity of the yearly influenza outbreaks that would be as good as the existing laboratory and sentinel surveillance.

## Methods

Web queries submitted to the web site Vårdguiden.se (www.vardguiden.se) were analysed. The web site is written in Swedish, thus the submitted queries are (mostly) in Swedish. Although the site is accessible by anybody, the primary users are residents in the Stockholm County [18]. However, as no information on the users submitting the queries was available, the data were aggregated on a national level. In the described study, we used Vårdguiden logs from June 27, 2005 to June 24, 2007, thus covering two influenza seasons. No spelling corrections were considered (these are provided by the search engine), nor did we remove possible duplicate searches where a user had submitted a query more than once. All queries were case-folded (that is, turned into lower case). The data were aggregated by week, which is the aggregation level for the sentinel and the laboratory reports. There were logs missing for in total five weeks during the summer of 2006, a period normally not affected by influenza in the northern hemisphere.

Two sets of reference data were used: the number of laboratory verified influenza cases and the proportion of patients with influenza-like illness having seen any of the sentinel general practitioners (GPs) in Sweden. The influenza season normally lasts from the end of November to mid April, with a peak sometime in February/March for most seasons. The reporting is done from October (week number 40) to May (week number 20).

In total, twenty types of queries were included in the statistical analysis. For examples of each type, see Table 1. More specifically, the analysis was performed on queries containing the word *influensa* (influenza in Swedish) in various variants and on queries on symptoms for influenza-like illness. The seven investigated symptoms were: fever, headache, myalgia, cough, sore throat, coryza, and shortness of breath. These symptoms were motivated by the following ILI-definition:
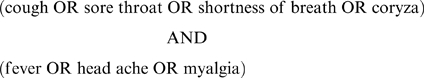



**Table 1:** Summary of investigated queries, with genuine examples in Swedish (one complete query per line) in addition to their English translations.

Query type	Explanation	Examples in Swedish	Examples translated to English	Total number of queries (% of all queries)
Influenza	A one word query containing “influenza”.	*Influensa*	*influenza*	5,745 (0.14)
		(the only matching query)		
Influenza in complex search	The word “influenza” as a single word in combination with something else.	*influensa hos barn*	*influenza in children*	1,366 (0.03)
		*influensa vaccination*	*influenza vaccination*	
Influenza as one word	The word “influenza” is present as a single word either as the only term or in a multi word query.	*influensa*	*influenza*	7,111 (0.17)
		*influensa hos barn*	*influenza in children*	
		*influensa vaccination*	*influenza vaccination*	
Influenza compound	The word “influenza” is part of a compound.	*influensavaccination*	*influenza vaccination*	7,054 (0.17)
		*maginfluensa*	*stomach flu*	
Influenza and more	The query contains “influenza” in any constellation.	*influensa*	*influenza*	14,165 (0.34)
		*influensa hos barn*	*influenza in children*	
		*fågelinfluensa*	*bird flu*	
		*influensavaccin vid graviditet*	*influenza vaccine during pregnancy*	
Cleaned influenza	The query contains “influenza”, but queries matching “bird”, “stomach flu” or “vaccination” are removed.	*influensa*	*influenza*	7,072 (0.17)
		*influensa hos barn*	*influenza in children*	
		*influensavaccin vid graviditet*	*influenza vaccine during pregnancy*	
Stomach flu	The query contains “stomach flu”.	*maginfluensa*	*stomach flu*	2,730 (0.06)
		*inkubationstid maginfluensa*	*incubation period stomach flu*	
Influenza and symptom	The query contains two or more ILI symptoms and possibly “influenza”.	*influensa hosta*	*influenza cough*	95 (0.00)
		*feber influensa*	*fever influenza*	
		*hosta snuva halsont*	*cough coryza sore throat*	
ILI	The query matches the given ILI definition; may also contain other words.	*hosta feber*	*cough fever*	448 (0.01)
		*hosta och feber*	*cough and fever*	
		*halsont ej feber*	*sore throat no fever*	
		*feber snuva ont i halsen*	*fever coryza sore throat*	
More than one ILI symptom	The query contains at least two ILI symptoms, regardless of the definition, without other terms.	*halsont huvudvärk*	*sore throat head ache*	289 (0.01)
		*huvudvärk feber*	*head ache fever*	
		*hosta snuva halsont*	*cough coryza sore throat*	
Cough	A one word query containing “cough”.	*hosta*	*cough*	5,646 (0.13)
		(the only matching query)		
Sore throat	A one word query containing “sore throat”.	*halsont*	*sore throat*	3,620 (0.09)
		(the only matching query)		
Shortness of breath	A one word query containing “shortness of breath”.	*andningsbesvär*	*shortness of breath*	186 (0.00)
		(the only matching query)		
Coryza	A one word query containing “coryza”.	*snuva*	*coryza*	385 (0.01)
		(the only matching query)		
Fever	A one word query containing “fever”.	*feber*	*fever*	9,338 (0.22)
		(the only matching query)		
Headache	A one word query containing “headache”.	*huvudvärk*	*headache*	4,575 (0.11)
		(the only matching query)		
Myalgia	A one word query containing “myalgia”.	*muskelvärk*	*myalgia*	385 (0.01)
		(the only matching query)		
Cough and more	The query contains “cough” in any constellation.	*hosta*	*cough*	1,037 (0.02)
		*hostmedicin*	*cough syrup*	
		*hosta på kvällen*	*cough in the evenings*	
Fever and more	The query contains “fever” in any constellation.	*feber*	*fever*	11,128 (0.26)
		*körtelfeber*	*glandular fever*	
		*feber spädbarn*	*fever infant*	
Cold	A one word query containing “cold”.	*förkylning*	*cold*	32,156 (0.76)
		(the only matching query)		

The table also shows the total number of queries for the two seasons, as well as the percentage of queries matching the query type.

This definition is based on the ECDC definition, but adapted to the data source, where “sudden onset” is difficult to identify. Also the ECDC definition is supposed to be used by a doctor, while such a person normally is not involved in the formulation of the web queries.

We further counted influenza matches cleaned from queries on items not related to ordinary influenza, counting only queries *not* containing the Swedish words *vaccin* (vaccine), *fågel* (bird), or *maginfluensa* (stomach flu). Nineteen per cent of the queries matching influenza were on stomach flu, why we also specifically included this query in the examined set. As for the queries on symptoms, in addition to counting these when being the only submitted word, we counted the number of queries matching the ILI definition given above, allowing for other terms in the query. The two most frequently occurring symptoms of the ILI-symptoms (fever and cough), were also investigated when occurring in any constellation. (The Swedish word for cough (*hosta*) loses its *a* when being the first element in a compound. This is accounted for in the program counting the occurrence.) Additionally, we examined the term cold (*förkylning*). All selected query terms consist of one word in Swedish. This is worth noting as about 75 per cent of the queries contain a single term only (Swedish is rich in compounding, and the Swedish equivalence to, for example, influenza vaccine is *influensavaccin*).

Since the usage of the search engine on Vårdguiden.se increases over time, data were standardised by dividing the counts during one season by the total number of queries to the web site during that particular season. The calculated numbers for the different types of Vårdguiden.se queries were highly correlated, which poses a problem for regular regression due to colinearity issues. Therefore, *partial least squares regression* (PLSR), which is an approach designed for these kinds of data, was used to generate our estimating models. This is a method used in many application areas for multivariate, highly correlated data [19]. PLSR works by “relating two data matrices, X and Y, to each other by a linear multivariate model” [20, p71]. This is done by using an algorithm closely related to *Principal Component Analysis*, to transform a highly dependent set of input data into a set of independent components.

**Figure 1:**
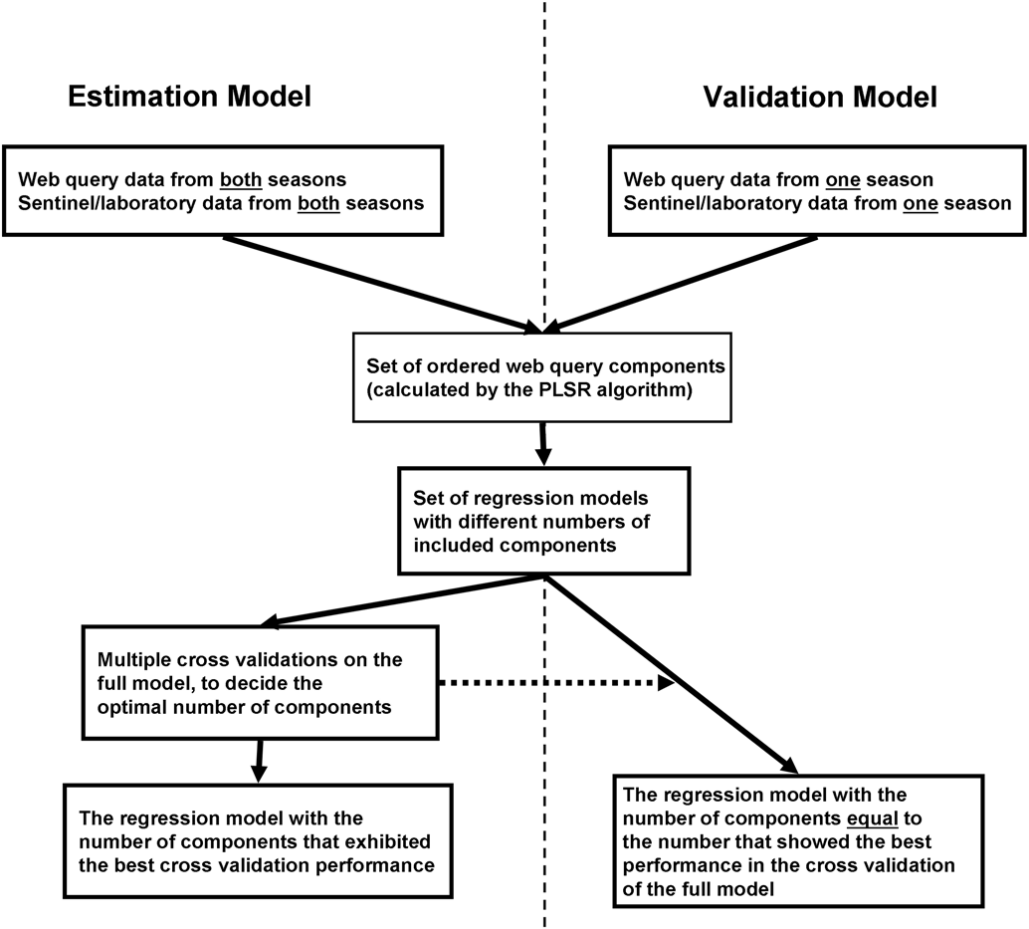
A flow-chart of the statistical analysis.

The entire PLSR procedure runs as follows (see also Figure 1): Given a set of outcome variables (Y) and a set of input variables (X) it creates new variables (“components”) by adding together the input variables in X, with individual weights for each variable. This is done as many times as there are input variables, with a different set of weights each time. The weights are chosen in such a way that a newly created component exhibits as much as possible of the variation in input and output data that has not been included in previous components. Thus, the first component to be created describes most of the variation in the data, the second describes a little less, and so on. The weights are also chosen so that each component is independent from the others, that is, a single component can not be described as the sum of the other components. Subsequently, the created components are used as input variables for a set of ordinary regressions predicting Y, with increasing number of components included. Finally, the models are validated, in order to establish the number of components to include. The model which exhibits the best validation performance is chosen as the final model. In our analysis the PLSR was applied by using the wide kernel algorithm, as implemented in the PLS package [21] in R 2.6.1 [22].

The sentinel and the laboratory values from both seasons were used as input variables to two different models, one model for each source, where both used all twenty types of queries as predictor variables.

In our experiments, we used cross validation [23] to find the optimal number of components to include in the models. Briefly, cross validating a model is done by first splitting the data set into a number of equally sized partitions. One partition is then omitted, while the remaining partitions are used to estimate a model. This model is subsequently used to predict the omitted data. Thereafter the difference between the true and the predicted value is measured. This process is repeated multiple times with different partitions omitted. Finally, all obtained differences are squared and averaged to generate an estimate of the precision of the cross validated model. We thus get the *mean predictive error*. When only one observation is omitted, the process is referred to as *leave-one-out cross validation*.

We conducted a number of different cross validations, where the number of partitions that the data was split into was varied, from two up to 60. In addition, we tested the extreme case, where only one week was omitted at a time. In the presented research, the omission was done sequentially, that is, we first omitted the first *n* weeks, then the second *n* weeks and so on. The resulting 60 different mean predictive errors were used to select the optimal model.

## Results

### Queries in total and queries on influenza

During the two examined seasons, there were in total 1,522,802 (2005/2006) and 2,699,097 (2006/2007) queries submitted to the search engine on the Vårdguiden web site. Of these queries, in total 1,737 (0.11 per cent) and 4,008 (0.15 per cent) consisted of only the Swedish word corresponding to influenza, for the two influenza seasons respectively. The influenza queries were thus a small fraction of all queries submitted. Raw data for the selected queries are displayed in Table 1, showing the total number of matching queries for the two investigated seasons, as well as the percentage of queries matching the query type.

### Relation between influenza surveillance and web queries

**Figure 2:**
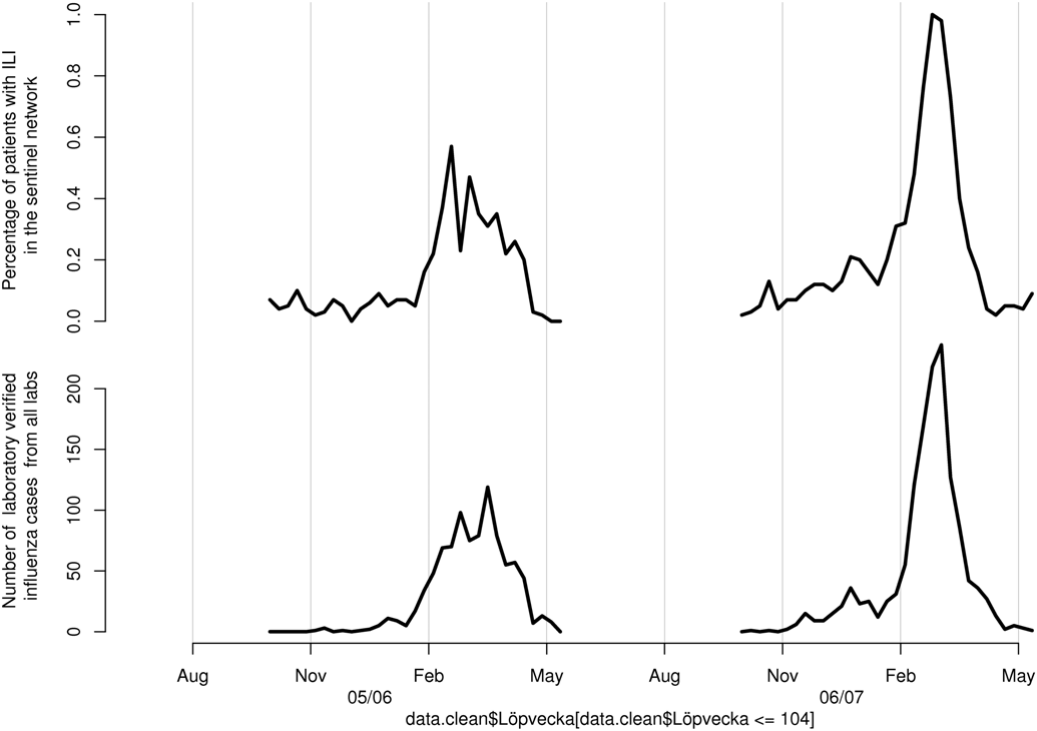
An overview of the sentinel and the laboratory data for the two investigated influenza seasons (2005/2006 and 2006/2007).

**Figure 3:**
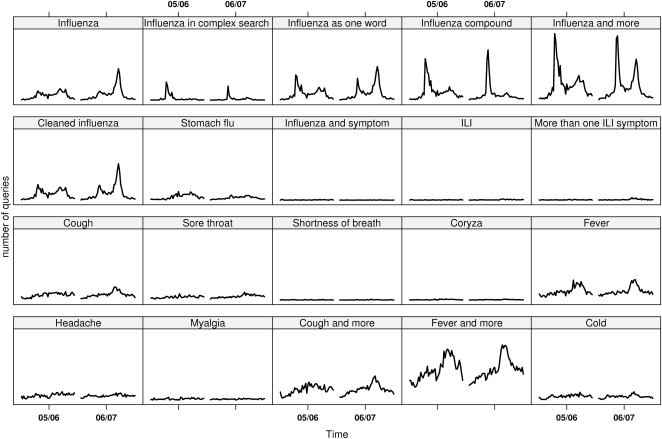
The number of queries matching the selected query types plotted over time.

Figure 2 gives an overview of the percentage of cases with ILI reported through the sentinel system as well as the number of laboratory verified cases. In Figure 3, the number of queries matching each one of the selected query types is plotted over time. As mentioned, the data were aggregated by week for the investigated period, covering two influenza seasons.

Most of the selected types of queries followed the same epidemic pattern as the sentinel and the laboratory influenza data. Those that did not had a low occurrence, making the assessment difficult. For many query types, two peaks can be discerned. If comparing at what time of the year these peaks occur, it is often at the same time as the influenza peaks (see Figure 2). This is also true for the queries on stomach flu, and we have seen in other surveillance systems at the Swedish Institute for Infectious Disease Control that the norovirus season tends to run in parallel with the influenza season. However, if visually inspecting the curves for the twenty query types, it can be noted that the queries on “stomach flu” show no difference for the two seasons, while the queries related to influenza follow the pattern of the laboratory and the sentinel data, with a stronger signal the second season.

The validation process showed that when comparing all the generated mean predictive errors for the different models, the model that included four components had the best (that is, the lowest) values in all but five versions of the cross validation for the laboratory data, and for all but sixteen versions for the sentinel data. In the versions where the four component model did not have the best performance, the model that included three components did. This occurred when large portions of the data were omitted, that is when there was a small amount of data from which to draw estimates. Based on these results, the four component model was selected as optimal for both the laboratory and the sentinel estimation. Table 2 shows the R2 values and the mean predictive errors using leave-one-out cross validation for all generated models for the sentinel and the laboratory models respectively.

**Table 2:** R2 values and mean predictive errors using leave-one-out cross validation for all generated models for the sentinel and the laboratory models respectively.

Number of components in model	R2 (sentinel model)	R2 (laboratory model)	Mean predictive error (sentinel model)	Mean predictive error (laboratory model)
1	0.76	0.78	0.12	26.43
2	0.84	0.84	0.09	22.14
3	0.88	0.88	0.08	20.00
4	**0.89**	**0.90**	**0.08**	**19.14**
5	0.90	0.90	0.08	19.64
6	0.90	0.91	0.09	20.26
7	0.90	0.91	0.09	19.61
8	0.90	0.92	0.09	20.12
9	0.90	0.92	0.09	20.79
10	0.90	0.92	0.09	21.14
11	0.90	0.92	0.10	21.35
12	0.90	0.92	0.10	21.17
13	0.90	0.92	0.10	21.03
14	0.90	0.92	0.10	20.97
15	0.90	0.92	0.10	21.48
16	0.90	0.92	0.10	21.38
17	0.90	0.92	0.10	21.32
18	0.90	0.92	0.10	21.27
19	0.90	0.92	0.10	21.50
20 (all)	0.90	0.92	0.10	21.50

The values for the chosen models (using 4 components) are marked in bold.

**Figure 4:**
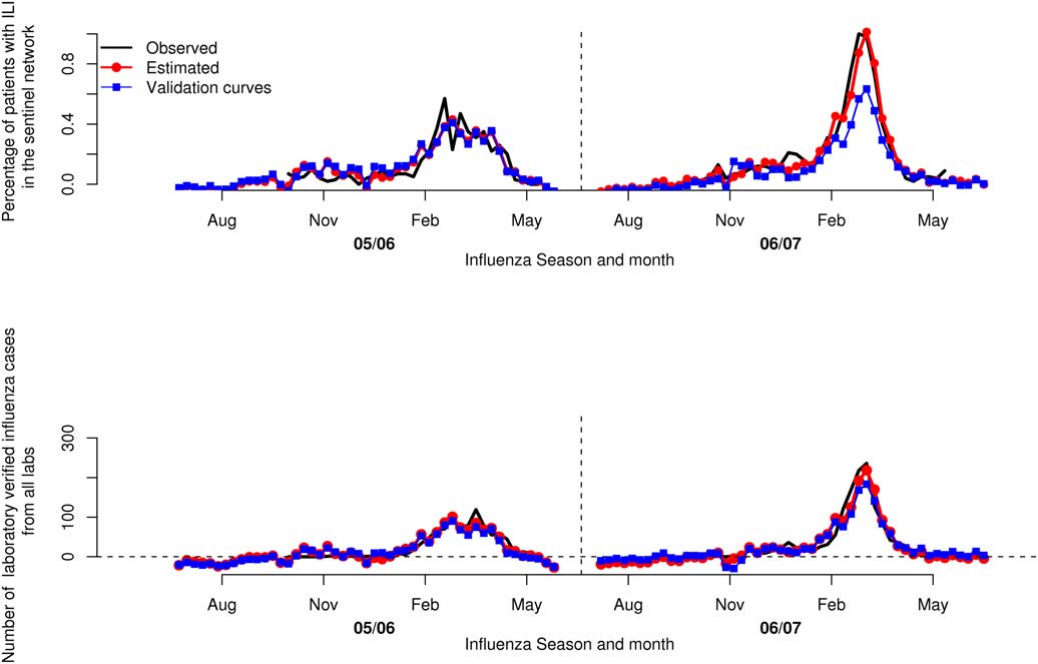
Observed values (black), predicted values from the full models (red, with circles), and predicted values using a model fitted on data from the opposite season (blue, with squares) for the model predicting sentinel values and for the model predicting laboratory values.

Estimations for the four component models are shown in Figure 4. The upper graph shows the estimated sentinel values and the lower graph shows the estimated laboratory values. The black line denotes the observed values and the red line with circles denotes the predicted values from the full models. As can be seen in this figure, the numbers estimated by the models ended up following the observed epidemic curves very closely indeed. Over the two seasons the obtained R2 values were 0.89 for the sentinel data and 0.90 for the laboratory data. This is only slightly less than the R2 values of 0.90 and 0.92 respectively for the models that included all 20 components but these models had larger mean predictive errors. Since this is the fit between the model and the data that were used to calibrate the same model, a close fit is to be expected. The laboratory curve differed by on average 19 verified cases, while the sentinel curve differed by on average 0.08 percentage points.

### Validation of results

The final predictive models encompass four components. In order to further validate the results, equivalent four-component models were fitted using a reduced data set with observations from only one season (see Figure 1). These models were then used to estimate the season that had not been used for fitting. This was done for both the sentinel and the laboratory data respectively. The results of this validation can also be seen in Figure 4, which shows the values predicted from these validation models, using data only from the opposite season (blue lines with squares). As can be seen, the “blindly” estimated values still follow the general trend of the observed data. When estimating the 2006/2007 season in this way, the estimated epidemic peak matched the observed peaks well, both with regards to the time span and, for the laboratory data, the intensity level. The result is not quite as good for the 2005/2006 season. This is probably because an intense season generates a stronger signal in the query stream than does a relatively mild season. In addition, the web site traffic grew from the 2005/2006 to the 2006/2007 season, meaning that the 2006/2007 season had more data on which to base the analysis.

### Loading weights of the queries

**Figure 5:**
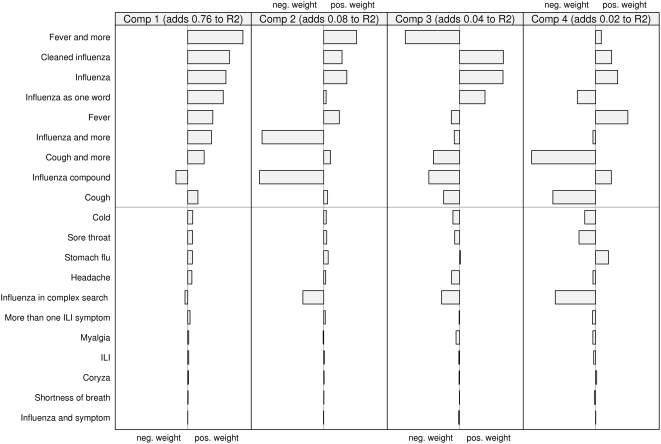
The relative contribution of the scaled and centred queries to each component in the model predicting sentinel values.

**Figure 6:**
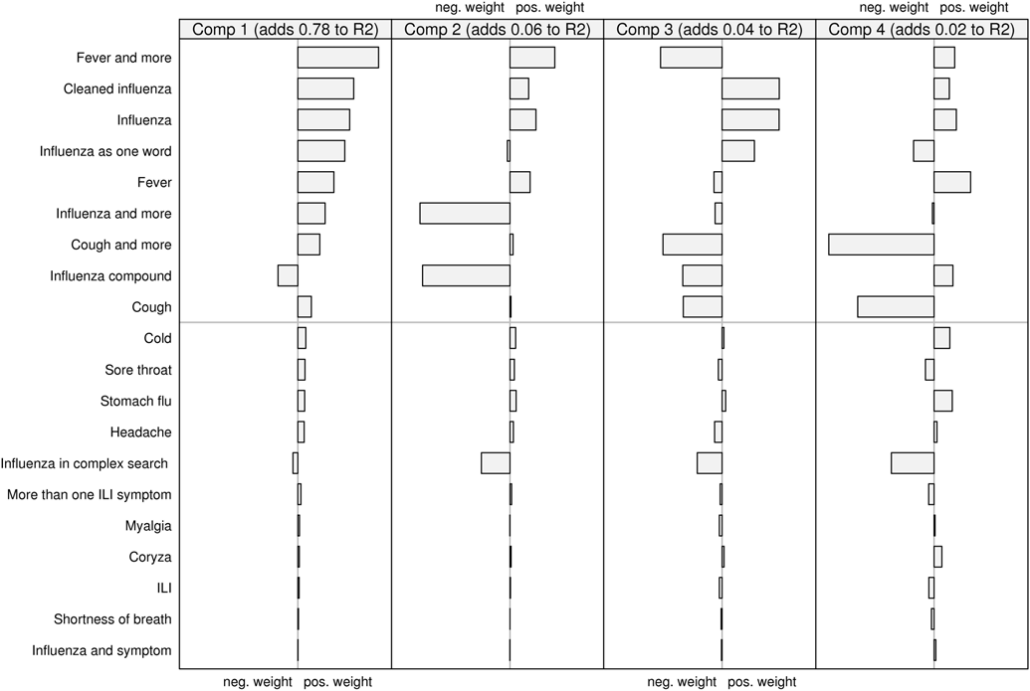
The relative contribution of the scaled and centred queries to each component in the model predicting laboratory values.

Looking at how the two models are composed also highlights some interesting features. In Figures 5 and 6, the relative contribution (the “loading weights”) of the scaled and centred queries to each component is shown. The first two components are dominant, together explaining about 84 percent of the variation for both laboratory and sentinel data. It can be seen that about half the query types seems to be important. The rest, those below the gray line in the figures, have very low weights. Fitting a model without these query types resulted in only a minimal change in the predicted curves. Looking at the upper set of queries, the first component is the sum of all queries except “influenza compound”. The second component is dominated by the negative weights of “influenza compound” and “influenza and more”. If looking at how the components are represented over time (not depicted in this paper) the second component has a great dip around October and November, which is the time of the year when the influenza vaccination campaigns are launched.

## Discussion

In the study reported in this paper, we investigated how well web queries submitted to a Swedish medical web site mimic the results from other systems for influenza surveillance, which in turn can describe the influenza prevalence in Sweden. We found that the intensity of certain web queries on influenza and influenza-like illness follows the same pattern as the laboratory and sentinel reports for influenza, and that they can be used as input data for accurate estimation models.

By using a statistical approach that takes various queries into account, a lot is gained compared to a simpler model. The main advantage is that more data can be used as the basis for the estimation, which naturally increases accuracy and decreases variability. In addition, by including queries that may reflect media coverage of, for example, bird flu, bias can be reduced. It should be noted that we need reference data in order to calibrate the model. In the influenza context, this means that an existing surveillance system is needed for a PLSR based model to be achievable. Also, because of the flexibility of the PLSR method, the model will mimic the characteristics of the surveillance system, perhaps hiding characteristics particular to web queries. In addition it must be noted that the PLSR model as applied does not assume any time dependency between the weeks, and can thus not generate any predictions about the future. A time aspect could possibly be introduced in future models. While using a nonparametric approach, such as a *K Nearest Neighbours* algorithm would have been feasible, these kinds of models are more difficult to interpret than a parametric model, and therefore less attractive in this context.

The search engine does not log any information on the users submitting the queries, not even the IP-address which theoretically could pin-point the user geographically. The data are therefore aggregated on a national level, meaning that only national outbreaks and trends can be discovered. In February 2008, Vårdguiden performed a survey directed to the visitors of the web site [18]. In total 1,224 persons were questioned, out of which 1,000 replied. According to this survey, 85.1 per cent of the visitors were women. The majority of all visitors were between 21 and 35 years old, and 63.4 per cent lived in the Stockholm County. Of the respondents, 74.3 per cent used the search engine on the web site to look for the information they require.

As timeliness may be crucial in the case of an outbreak, sources conveying as early outbreak indicators as possible should be identified. Zeng & Wagner have constructed a model ― based on health psychology literature ― on people's behaviour when falling ill [24]. The model consists of four phases: *recognition of symptoms*, *interpretation of symptoms*, *cognitive representation of illness*, and *seeking treatment*. The model implies that outbreak indicators are found in different sources depending on which phase the infected persons are in. For the first phase, one potential source are records of absenteeism. During the second and third phases, the social surrounding plays an important role. Also, ill people may seek advice on medical help lines and web sites during these two phases. The model shows the natural delay between noting of symptoms and seeking treatment by, for example, visiting a pharmacy or a health centre, which is done only during the fourth phase. According to Zeng & Wagner the queries will reflect a person's symptoms when in the second phase (interpretation of symptoms), while during the third phase (cognitive representation of illness) the search terms are more disease oriented. Consequently, the queries reflecting symptoms should be an even earlier indicator than queries reflecting diagnosis. In theory, this model probably has great bearing, but the acuteness of an influenza illness means that it is difficult to discern the phases with the time resolution used in this study. However, if the queries could be analysed momentaneously, such an approach would probably allow for a more accurate representation of the epidemic pattern.

Andersson et al. have shown that the sentinel reports cannot be used to predict the number of laboratory verified cases in the Swedish data set [17]. This fact is a strong argument for complementing the influenza surveillance with analyses based on other data sources. By ― as in this study ― using web query data, it is also possible to obtain a baseline for the time of the year when no conventional influenza surveillance is performed. In addition, public holidays such as Christmas, when seeing a GP is more difficult, are covered with this source. Assumingly, different strata of the population are covered by the web queries, compared to those covered by the reporting from the health care. The use of the internet is increasing, and we can therefore expect to get more and more data points, and consequently more reliable results. With time, the internet will be an integrated part of most citizens' life and for this reason we will also get a much more diverse group of users. Thus, the web queries have the potential to reflect the population as a whole. This is opposed to the sentinel system as well as the laboratory testing, which probably over-report groups that are more vulnerable to the disease.

With the presented study we show that web queries can indeed be a reliable and cost-effective source for identification and estimation of the development of the aggregated influenza activity in a society. We feel that this first, scientific evaluation of its usefulness is an important proof of concept that could stimulate further investigations, and also an important foundation for reliable exploitation of similar systems for various kinds of surveillance. Web query logs are obtained at a low cost, and give a unique access to ill individuals who may yet not be seeking care. This paper shows the potential of web queries as an accurate and labour extensive source for syndromic surveillance. In future work, we will explore how to best utilize the web queries for other diseases, with characteristics different from influenza, such as norovirus infections.
